# Topical Ectoine: A Promising Molecule in the Upper Airways Inflammation—A Systematic Review

**DOI:** 10.1155/2019/7150942

**Published:** 2019-08-26

**Authors:** Manuele Casale, Antonio Moffa, Samanta Carbone, Francesca Fraccaroli, Andrea Costantino, Lorenzo Sabatino, Michele Antonio Lopez, Peter Baptista, Michele Cassano, Vittorio Rinaldi

**Affiliations:** ^1^Unit of Otolaryngology, UOS ORL TI, Campus Bio-Medico University, Rome, Italy; ^2^Unit of Otolaryngology, University of Foggia, Foggia, Italy; ^3^Unit of Otolaryngology, Clinica Universitaria de Navarra, Pamplona, Spain

## Abstract

To date, topical therapies guarantee a better delivery of high concentrations of pharmacologic agents to the mucosa of the upper airways (UA). Recently, topical administration of ectoine has just been recognized as adjuvant treatment in the Allergic Rhinitis (AR) and Rhinosinusitis (ARS). The aim of this work is to review the published literature regarding all the potential therapeutic effects of ectoine in the acute and chronic inflammatory diseases of UA. Pertinent studies published without temporal limitation were selected searching on MEDLINE the following terms: “ectoine” and “nasal spray,” “oral spray,” “upper respiratory tract infections,” “rhinosinusitis,” “rhinitis,” “rhinoconjunctivitis,” “pharyngitis,” and “laryngitis.” At the end of our selection process, six relevant publications were included: two studies about the effect of ectoine on AR, one study about ARS, one study about rhinitis sicca anterior, and two studies about acute pharyngitis and/or laryngitis. Due to its moisturizing and anti-inflammatory properties, topical administration of ectoine could play a potential additional role in treatment of acute and chronic inflammatory diseases of UA, in particular in the management of sinonasal conditions improving symptoms and endoscopic findings. However, these results should be viewed cautiously as they are based on a limited number of studies; some of them were probably underpowered because of their small patient samples.

## 1. Introduction

Ectoine is a low-molecular, cyclic tetrahydropyrimidine organic osmolyte, which was first identified by Galinski et al. [1] in the halophilic bacterium, Ectothiorhodospira halochloris, but since then it has been found in other extremophiles microorganisms. To protect themselves from external stress factors such as extreme temperatures, high salt concentrations, and ultraviolet radiations, these microorganisms produce stress-protection molecules, also called extremolytes. Among these molecules, there is ectoine [2]. It is known that ectoine works via an entropy-driven mechanism called “preferential exclusion” or “preferential hydration” during which ectoine influences the characteristics of the “water shell” surrounding biomolecules like membranes. By excluding osmolytes from the direct hydrate shell of proteins and membranes, a preferential hydration of such proteins or membranes occurs, thereby stabilizing their native confirmation and making them less vulnerable to external stressors. As suggested by Yancey PH [3] and Arakawa T. et al. [4] destabilizers molecules such as some salt ions and urea generally bind to proteins, causing them to unfold because this exposes more groups that undergo thermodynamically favorable binding with the destabilizer. By contrast, many stress-protection molecules such as ectoine do not bind to proteins; indeed, they are excluded from a protein's hydration layer (the water molecules adjacent to a protein's surface). Because of this repulsion, proteins will tend to fold up more compactly, since this will reduce exposure of the peptide-bond backbone to thermodynamically unfavorable interactions with the stabilizing solute.

In addition to its moisturizing properties, it has been shown that ectoine limits the inflammatory cascade at the membrane level of respiratory and skin cells. In particular, patients' pulmonary epithelium affected by Chronic Obstructive Pulmonary Disease, ectoine is able to reduce the neutrophilic inflammatory response induced by exposure to environmental and occupational pollutants such as the carbon nanoparticles [5]. Although the precise mechanisms are not completely clear, it seems that this molecule plays a stabilizing role in receptor structures, effectively preventing the signalling cascade [6]. Taken together, anti-inflammatory and hydration properties make ectoine an interesting candidate for application in diseases where cell membrane protection is fundamental. Indeed, ectoine is widely used in dermatology and neither contraindications nor interactions with drugs are reported. Topical application of ectoine to lesional skin of patients suffering from mild to moderate atopic dermatitis significantly reduced the clinical severity of this disease [7]. Recently, topical administration of ectoine has also been used for many inflammatory diseases' treatment of the upper airways (UA) such as Allergic Rhino Conjunctivitis (ARC), Rhinitis Sicca Anterior, and Acute Pharyngitis. A 2014 meta-analysis [8] concluded that the use of ectoine-based nasal sprays improves symptoms of ARC with no side effects, effectively reduces sinonasal symptoms, and represents an exciting alternative in the management of ARC. However, this paper included only three studies, two of them not published before, without detailed information, excluding clinical trials about the use of ectoine on other upper airways diseases.

The aim of this work is to review the published literature regarding all the potential therapeutic effects of ectoine in the acute and chronic inflammatory diseases of UA, trying to investigate better the main application areas of ectoine, the root and schedule of ectoine administration, and the main efficacy parameters evaluation.

## 2. Methods

### 2.1. Search Strategy and Article Selection Process

A search was made of the PubMed, Google Scholar, and Ovid databases according to the PRISMA guidelines [9] using the following key words or in the case of PubMed medical subject headings: “ectoine” and “nasal spray,” “ectoine” and “oral spray,” “ectoine” and “upper respiratory tract infections,” “ectoine” and “allergic rhinitis,” “ectoine” and “rhinosinusitis,” “ectoine” and “rhinoconjunctivitis,” “ectoine” and “rhinitis,” “ectoine” and “pharyngitis,” ectoine” and “laryngitis.” Only studies in English, published in peer-reviewed journals, reporting data on the role of the topical administration of ectoine in the UA are included; no studies related to dentistry have been considered. Literature reviews, technical notes, letters to editors, and instructional course were excluded. Additional literature was found by reviewing the reference lists of the selected articles. Hence, the authors then independently assessed the full-text versions of each publication and excluded those whose content was judged not to be strictly related to the subject of this review.

At the end of our selection process, we included six clinical studies which investigated the potential role of topical ectoine in UA, involving 474 patients: two studies about AR, one study about ARS, one study about rhinitis sicca anterior, and two studies about acute pharyngitis and/or laryngitis.

## 3. Results

The effects of these studies are summarized in Table 1.

### 3.1. Ectoine and Allergic Rhinitis

Sonnemann U et al. [10] compared the effect of ectoine nasal spray with beclomethasone nasal spray on 50 patients with AR for 14 days. In particular, 25 patients used 2% ectoine nasal spray (three times a day) while 25 patients used 0.05% beclomethasone nasal spray (twice a day). During the treatment period, patients and physicians evaluated total nasal symptom score (TNSS), single symptoms score (nasal obstruction, rhinorrhea, nasal itching, and sneezing), efficacy, and tolerability of both treatments. Patients also completed a quality of life questionnaire. At the end of the treatment, physician's assessment of TNSS showed a significant reduction from baseline in both groups while patient's assessment showed a significant reduction only for beclomethasone group. The authors did not confirm the noninferiority of ectoine compared to beclomethasone nasal spray. Only from single symptoms patient's analysis, a significant reduction of sneezing in beclomethasone group was recorded compared to ectoine group. There were not any significant differences between both groups for patient's evaluation of quality of life, while in the physician's evaluation a significant improvement for the frequency of brushing the nose in beclomethasone was observed compared to ectoine group. Finally, patient and physicians judged the tolerability of both products similarly (“good” to “very good”) without differences. Efficacy assessed by patients and physician significantly increased over the treatment period in beclomethasone group compared to ectoine group.

Werkhauser N et al. [11] investigated the effects of ectoine products in patients with AR in two studies. In the first trial, 48 patients were included: 22 received ectoine eye drops and nasal spray (one eye drop and one puff of nasal spray four times per day) and 26 patients received azelastine nasal spray and eye drops (one eye drop and one puff of nasal spray twice per day) for 7 days. Patients and physicians evaluated TNSS (nasal obstruction, rhinorrhea, nasal itching, and sneezing) and total ocular symptom score (TOSS) (conjunctivitis, eye itching, and tearing), efficacy, and tolerability of both treatments. Only physicians judged compliance. At the end of the treatment, TNSS and TOSS evaluated by physicians showed a significant reduction from baseline in both groups. TNSS and TOSS evaluated by patients decreased from baseline in both groups without significant difference. Patients and physicians assessments of efficacy and tolerability of both groups were similar without significant difference. Compliance was good in both groups, with no significant difference.

In the second study, 50 patients were included: 25 received ectoine nasal spray (five times per day) whereas 25 received cromoglycic acid nasal spray (four times a day). After 7 days, patients swapped to the other treatment. During the follow-up visits physicians and patients assessed TNSS, TOSS, efficacy, and tolerably of both treatments. Compliance was judged only by physicians. TNSS evaluated by physicians and patients significantly decreased in both groups for all 14 days' treatment. TOSS evaluated by patients significantly decreased for all 14 days only in group with ectoine spray while TOSS evaluated by physicians significantly improved for all 14 days in both groups.

According to the physicians and patients judgment, the efficacy of both treatments was rated “good to satisfactory” without significant differences. Tolerability judged by physicians and patients was significantly better following a 7-day treatment with ectoine containing nasal spray in comparison to cromoglycic acid product. The compliance was assessed as very good by the physician, and values were not statistically different between groups.

### 3.2. Ectoine and Acute Rhinosinusitis

Eichel A et al. [12] compared the effects of 2% ectoine nasal spray to systemic treatment with herbal formula, in 66 patients with ARS for 14 days. In particular, 48 patients received ectoine nasal spray (one or two puffs into each nostril several times a day) and 18 patients received herbal phytotherapeutic dragées (one dragéet three times a day). During the treatment, physicians and patients recorded the Sinusitis Symptom Score (SSS) and adverse events. In addition, physicians performed nasal endoscopy and patients completed a sinusitis-specific health related quality of life (HRQL) questionnaire. At the end of the treatment, the SSS evaluated by physicians and patients decreased significantly from baseline during the study in both groups (58.1% in ectoine group and 53.5% in control group) without significant differences. Nasal endoscopy and HRQL questionnaire scores improved from baseline in both groups but significant difference was recorded only in ectoine group. No adverse effects have been reported.

### 3.3. Ectoine and Rhinitis Sicca

Sonnemann U et al. [13] evaluated the efficacy of ectoine nasal spray in patients with rhinitis sicca in two studies. In the first clinical trial, 50 patients used 0.5% ectoine nasal spray while in the second trial 30 patients used nasal spray containing 0.5% ectoine with 1.0% dexpanthenol. No control groups were expected. Patients and physicians assessed the main symptoms such as nasal obstruction and crusting of the nose and secondary symptoms such as endonasal blood deposits, concomitant pharyngitis, cacosmia, rhinorrhea, exudate viscosity, and turbinate hyperplasia. In addition, they judged efficacy and tolerability of both products. After 14-day treatment, nasal obstruction and nasal crust formations evaluated by physicians and patients improved significantly in both trials. Secondary symptoms evaluated by physicians significantly improved over the time in both studies, with the exception of rhinorrhea, which improved significantly only in the second study. From the analysis of the secondary symptoms conducted by patients, rhinorrhea significantly ameliorated in the first study while in the second study a significant reduction for bleeding, cacosmia, and exudate viscosity was also observed. Efficacy and tolerability were judged “good” by physicians and patients in both treatments.

### 3.4. Ectoine and Acute Pharyngitis and/or Laryngitis

Müller D et al. [14] compared the effects of ectoine oral spray to saline lozenges in the treatment of acute pharyngitis and/or laryngitis in 95 patients for 7 days. In particular, 64 patients received ectoine oral spray (one to two puffs several times a day) while 31 patients received lozenges (one to two lozenges up to six times a day). At all follow-up visits, physicians and patients evaluated hoarseness, swallowing difficulties, pharyngitis symptom score, tolerability, efficacy, and adverse events. At the end of the treatment, ectoine oral spray guaranteed an earlier improvement of hoarseness, swallowing difficulties, and pharyngitis symptom score than control group without statistical differences. Treatment with ectoine oral spray showed higher efficacy than control group, as judged by physicians and patients recording a statistical significance only in the patient's evaluation. Both physicians and patients rated the tolerability of the spray and the lozenges as “good” to “very good.” No serious adverse effects have been reported.

Dao VA et al. [15] enrolled 90 patients with acute pharyngitis: 35 patients using ectoine (1-2 lozenges every 3 h or as needed), 35 patients with hyaluronic acid (one or two lozenges every 2-3 hands up to six times daily if necessary), and 20 patients with hypertonic saline gargle (3-5 times daily). All treatment lasted 7 days. Physicians and patients assessed primary variables (pain on swallowing, urge to cough, and hoarseness), secondary variables (dry mouth and throat, reddening of the oropharynx, reddening of the larynx, burning sensation in the throat, and patient's general health condition), effectiveness, tolerability, and compliance. At the end of the treatment, primary variables significantly decreased in all three groups from baseline. The reductions were significantly greater in ectoine and hyaluronic acid groups than saline gargle group. Regarding individual symptoms evaluated by physicians ectoine was more effective than hyaluronic acid in ameliorating the symptom of reddening of the larynx. Investigators and the patients confirmed that ectoine was significantly more effective than saline gargle in improving general health condition, but hyaluronic acid was not. Effectiveness evaluation conducted by physicians and patients showed a greater improvement in ectoine and hyaluronic acid groups than saline gargle group. The tolerability of ectoine was better than hyaluronic acid reaching significance only in the physicians' evaluation. Physicians' assessment of compliance showed significantly better value in ectoine group than hyaluronic acid group while patients' evaluation only highlighted that both lozenges were significantly better than the saline gargle.

## 4. Discussion

**Figure 1:**
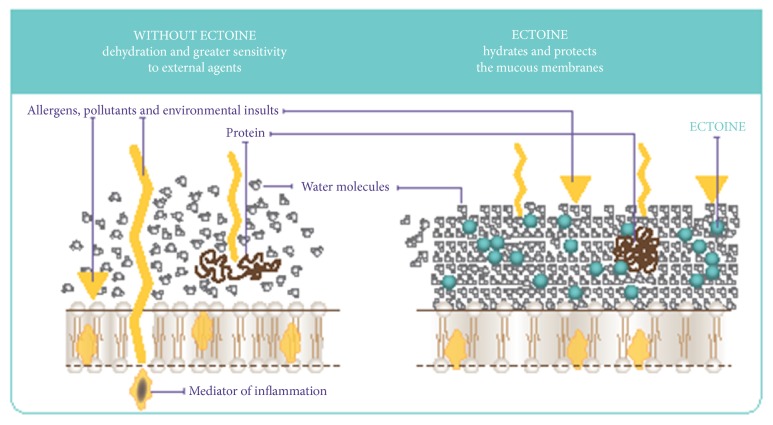
Ectoine's mechanism of action. Influence of water molecules alone (on the left) and aqueous solution of ectoine (on the right) on lipid bilayer.

Medical management of sinonasal and oral inflammatory diseases increasingly involves the use of topical agents, which offer an improved ability to deliver high concentrations of drugs to the UA mucosa avoiding systemic effects. Given the pivotal role that topical therapies play in many sinonasal and oral conditions, considerable interest in the effectiveness, tolerability, and compliance of specific new substances has become evident [16]. Literature data that emerged from our analysis allow us to affirm that topical administration of ectoine can be useful as coadjutant treatment in the acute and chronic inflammatory diseases of UA such as AR, ARS, rhinitis sicca, pharyngitis, and laryngitis. Ectoine is natural amino acid derivate produced by bacteria living under extreme environment conditions acting as osmoregulatory compatible solute. The stabilizations effects on the barrier function of the epithelial tissue have led to the hypothesis that ectoine increases the resistance of the UA mucosa and improves its recovery (Figure 1). Thanks to the “preferential hydration,” ectoine reduces nasal symptoms of patients with rhinitis sicca increasing the fluidity of the nasal epithelia and inhibiting the potential loss of water [13]. Topical ectoine has proved effective also for the most common inflammatory conditions of the pharynx and larynx, improving both the patients' diary data and the physical examinations, supporting a potential therapeutic role of ectoine on all the upper aerodigestive tract [14, 15]. In addition, Sonnemann U et al. [10] suggested ectoine nasal sprays as interesting alternative treatment strategies for symptom reduction in AR patients, particularly for those seeking nonpharmaceutical treatments, as they contain a natural substance and are free of preservatives. Ectoine could play a key role in allergic disease of the upper and lower airway reducing the exacerbation of the immune response in antigen-exposed sensitized individuals and allergic sensitization against daily life. In particular, it has been shown that ectoine effect on the exacerbation of the immune response is not due to an interaction with the external particles, which might reduce the reactive surface area and thereby diminish the proinflammatory effects. Instead, prevention of carbon nanoparticles induced lung inflammation represents an ectoine-dependent reaction of the organism thought a mechanism involving the stabilization of macromolecules located at the outer cell surface. This hypothesis is further supported by the finding that ectoine is able to reduce pathogenic endpoints in skin epithelial cells induced by solar UVA radiation as a source for physical stress due to a stabilization of lipid microdomains (rafts) in the cytoplasm membrane and a decrease in UVA-induced ceramide release in human keratinocytes [17–19]. It has also been shown that ectoine can reduce the allergic sensitization. In particular, carbon nanoparticles seem to enhance the migration of antigen loaded dendritic cells to the draining lymph nodes and ectoine appears to prevent this effect either via direct action on dendritic cells or via the suppression of the neutrophilic inflammation [20]. All these mechanisms might stabilize mucous membranes improving the epithelial barrier such as lining epithelia of the nose, thereby protecting those from invading allergens and reducing allergen-induced inflammations.

In all the trials analyzed, spray represents the most common device used and no serious adverse effects and contraindications have been reported. The clinical trials lasted between seven and fourteen days and ectoine nasal sprays have been used with a very different treatment frequency (from three to several times a day).

There are some lines of evidence that topically administered ectoine improves the global subjective and clinical status of patients affected by UA inflammatory diseases, even if these findings should be viewed cautiously as they are based on a limited number of studies, some of which were probably underpowered because of their small patient samples. Further researches should be performed in order to confirm the effectiveness of topical administered ectoine in such patients, to define the better therapeutic protocols (way of administration and dosage) and to selectively test its effectiveness in the paediatric age. The strong connection between particle diameter and site of the high concentration of nebulized particles in the UA suggests that it should be also mandatory to carefully choose the “ideal” nebulizer device to get better therapeutic results [21].

## Figures and Tables

**Table 1:** Effects of included studies.

Authors	Study design	Patients distribution	Mean age (years)	Disease	Therapy	Efficacy parameters	Results	Side effects
Uwe Sonnemann et al.	Controlled, open label, noninterventional, multicenter	50 patients: *ectoine group* (25); *beclomethasone group* (25)	33.3	Allergic Rhinitis	*Ectoine group*: isotonic solution containing 2% ectoine, natural salt and water, one puff into the each nostril three times a day. *Control group*: 0.05 mg beclomethasone dipropionate spray, two puffs twice a day. Both treatments were applied for two weeks.	Patients and physicians evaluated *total nasal symptom score (TNSS), single symptoms score* (nasal obstruction, rhinorrhea, nasal itching and sneezing), *efficacy and tolerability* of both treatments. Patients also completed a *quality of life questionnaire.*	Both treatments resulted in a significant decrease of TNSS values. Importantly, tolerability results were comparably good in both groups. Both treatments resulted in a clear improvement in the quality of life as assessed by a questionnaire answered at the beginning and at the end of the trial.	Three adverse events were reported: two cases of headache in the ectoine group (unlikely related to ectoine) and one case of “burning of nose” in the beclomethasone group (judged as probable).

Nina Werkhauser et al. *First study*	Controlled, noninterventional	48 patients: *ectoine group* (22); *azelastine group* (26)	35	Allergic Rhinitis	*Ectoine group:* one eye drop per eye and one puff of the nasal spray per nostril four times per day. *Azelastine group:* one eye drop per eye and one puff of the nasal spray per nostril twice per day. The treatment period was one week.	Patients and physicians evaluated *total nasal symptom score (TNSS) and total ocular symptom score (TOSS), efficacy and tolerability* of both treatments; only physicians judged *compliance.*	TNSS and TOSS evaluated by physicians showed a significant reduction from baseline in both groups. TNSS and TOSS evaluated by patients decreased from baseline in both groups without significant difference. Patients and physicians assessment of efficacy and tolerability of both groups were similar without significant difference. Compliance was good in both groups, with no significant difference.	Eight adverse events: two cases of burning of eyes and itching of throat in the ectoine group, and six (four cases of burning of eyes, one case of nausea and one case of headache) occurred in the azelastine group. No serious adverse events occurred.

Nina Werkhauser et al. *Second Study*	Controlled, noninterventional, cross-over trial	50 patients: *ectoine* group (25);*cromoglicic acid* group (25)	34	Allergic Rhinitis	*Ectoine group:* nasal spray had to be applied at least 5 times per day. *Cromoglicic acid group: *spray had to be applied 4 times a day. After one week, patients swapped to the other treatment, for another week.	Patients and physicians evaluated *total nasal symptom score (TNSS) and total ocular symptom score (TOSS), efficacy and tolerability* of both treatments; only physicians judged *compliance.*	According to the physicians and patients judgment, the efficacy of both treatments was rated “good to satisfactory” without significant differences. Tolerability judged by physicians and patients was significantly better following a 7-day treatment with ectoine containing nasal spray in comparison to cromoglicic acid product. The compliance was assessed as very good by the physician, and values were not statistically different between groups.	No adverse events were observed during treatment with ectoine containing nasal spray. In contrast, 13 patients complained about a burning sensation during treatment with cromoglicic acid nasal spray. No serious adverse events occurred.

Andrea Eichel et al.	Prospective, observational, open-label, controlled, nonrandomized	66 patients: *Ectoine group*(48); *herbal phytotherapeutic dragèet group* (18)	Ectoine group (52.9); Control group (55.0)	Acute rhinosinusitis	*Ectoine group:* received ectoine (one or two puffs into each nostril several times a day). *Control group:* received one drag*è*et three times a day. The treatment lasts two weeks.	Physicians and patients recorded the *Sinusitis Symptom Score (SSS)* and *adverse events.* In addition physician performed *nasal endoscopy* and patients completed a *sinusitis-specific health related quality of life (HRQL) questionnaire.*	SSS evaluated by physicians and patients decreased significantly from baseline without significant differences. Nasal endoscopy and HRQL questionnaire scores improved from baseline in both groups but significant difference was recorded only in ectoine group.	One single adverse event in the ectoine group: burning sensation in the nose and secretion (assessed as ‘likely'). No serious adverse events occurred.

Uwe Sonnemann et al.	Prospective, open-label, noncontrolled, noninterventional	50 patients were treated with ectoine nasal spray (containing 0.5% ectoine).	40.12	Rhinitis sicca	During two weeks of treatment, patients were asked to use the nasal sprays at least five times daily.	Patients and physicians assessed the *main nasal symptoms* such as nasal obstruction and crusting of the nose and *secondary nasal symptoms* such as endonasal blood deposits, concomitant pharyngitis, cacosmia, rhinorrhea, exudate viscosity, and turbinate hyperplasia. In addition, they judged *efficacy and tolerability* of both products.	Nasal obstruction and nasal crust formations evaluated by physicians and patients improved significantly. Secondary symptoms evaluated by physicians significantly improved over the time in both studies, with the exception of rhinorrhea, which improved significantly only in the second study. From the analysis of the secondary symptoms conducted by patients, rhinorrhea significantly ameliorated. Efficacy and tolerability were judged as good by physicians and patients.	One adverse event (judged as unlikely). No serious adverse events occurred.

Uwe Sonnemann et al. *Second trial*	Prospective, open-label, noncontrolled, noninterventional	30 patients were treated with nasal spray with 0.5% ectoine and 1.0% dexpanthenol.	39.80	Rhinitis sicca	During two weeks of treatment, patients were asked to use the nasal sprays at least five times daily.	Patients and physicians assessed the *main nasal symptoms* such as nasal obstruction and crusting of the nose and *secondary nasal symptoms* such as endonasal blood deposits, concomitant pharyngitis, cacosmia, rhinorrhea, exudate viscosity, and turbinate hyperplasia. In addition, they judged *efficacy and tolerability* of both products.	Nasal obstruction and nasal crust formations evaluated by physicians and patients improved significantly. Secondary symptoms evaluated by physicians significantly improved over the time in both studies, with the exception of rhinorrhea, which improved significantly only in the second study. From the analysis of the secondary symptoms conducted by patients a significant reduction for bleeding, cacosmia, and exudate viscosity was also observed. Efficacy and tolerability were judged as good by physicians and patients.	One adverse event (judged as unlikely). No serious adverse events occurred.

Müller D et al.	Prospective, controlled observational, nonrandomized	95 patients: *ectoine oral spray* (64); *control group* (31)	Ectoine oral spray (50.53 ± 18.39); control group (47.1 ± 19.87)	Acute pharyngitis and/or laryngitis	*Ectoine oral spray:* one to two puffs several times a day. *Control group:* one to two lozenges up to six times a day. The treatment lasts one week.	At all follow-up visits, physicians and patients evaluated *hoarseness, swallowing difficulties, pharyngitis symptom score, tolerability, and efficacy.*	Ectoine oral spray guaranteed an earlier improvement of hoarseness, swallowing difficulties and pharyngitis symptom score than control group without statistical differences. Ectoine oral spray showed higher efficacy than control group, as judged by physicians and patients recording a statistical significance only in the patient's evaluation. Both physicians and patients rated the tolerability of the spray and the lozenges as ‘‘good” to ‘‘very good.”	In the ectoine group five adverse events: three were unlikely to be related, one was unrelated, and one was the relationship to the treatment medication that could not be evaluated. In the control group, one adverse event, which was unlikely to be related. No serious adverse event was reported.

Dao VA et al.	Prospective, active- controlled clinical study	90 patients: *ectoine group* (35), *hyaluronic acid group* (35) and *hypertonic saline gargle* (20).	Ectoine group (33.4 years), hyaluronic acid group (33.7 years), saline gargle group (49.4 years)	Acute pharyngitis	*Ectoine group*: 1-2 lozenges every 3 h or as needed; *Hyaluronic acid group:* one or two lozenges every 2-3 hands up to six times daily if necessary; *Hypertonic saline gargle group*: 3-5 times daily. All treatment lasted 7 days.	Physicians and patients assessed *primary variables* (pain on swallowing, urge to cough, and hoarseness), *secondary variables* (dry mouth and throat, reddening of the oropharynx, reddening of the larynx, burning sensation in the throat, and patient's general health condition), *effectiveness, tolerability and compliance.*	Primary variables significantly decreased in all three groups from baseline. The reductions were significantly greater in ectoine and hyaluronic acid groups than saline gargle group. Ectoine was more effective than hyaluronic acid in ameliorating the reddening of the larynx. Ectoine was significantly more effective than saline gargle in improving general health condition. Effectiveness evaluation showed a greater improvement in ectoine and hyaluronic acid groups than saline gargle group. The tolerability of ectoine was better than hyaluronic acid reaching significance only in the physicians' evaluation. Physicians' assessment of compliance showed significantly better value in ectoine group than hyaluronic acid group while patients' evaluation only highlighted that both lozenges were significantly better than the saline gargle.	No serious adverse event was reported.
